# Mechanical properties and freeze–thaw durability of expansive soil reinforced with polypropylene and lignin fibers

**DOI:** 10.1371/journal.pone.0356058

**Published:** 2026-08-14

**Authors:** Liang He, Song Wang, Jinsong Zhang, Xiaofan Zhu, Changwei Zhou

**Affiliations:** 1 China Coal Technology & Engineering Group Nanjing Design & Research Institute Co., Ltd., Nanjing, China; 2 School of Civil Engineering and Architecture, Anhui University of Science and Technology, Huainan, China; University of Sharjah, UNITED ARAB EMIRATES

## Abstract

To address the deterioration of expansive soil in seasonally frozen regions subjected to freeze–thaw cycles, this study investigated the effects of fiber modification by incorporating polypropylene fiber (PP) and lignin fiber (LF) on the swelling behavior, mechanical properties, and freeze–thaw durability of expansive soil. Using expansive soil as the base material, the modification effects of individual fiber incorporation were evaluated through free swell ratio, no-load swell ratio, and direct shear tests. Freeze–thaw cycle tests were then conducted on the composite fiber-modified soils, and the microscopic mechanism was analyzed in combination with SEM observations. The main conclusions are as follows: (1) For single-fiber modification, lignin fiber (LF) at a dosage of 1.2% exhibited the best overall performance, producing the greatest reduction in free swell ratio and the largest increase in shear strength. The incorporation of polypropylene fiber (PP) also significantly improved these parameters, although its effect was weaker than that of lignin fiber (LF). (2) Based on the results of single-fiber modification, the addition of 1.2% polypropylene fiber (PP) on the basis of 1.2% lignin fiber (LF) further improved the mechanical properties of expansive soil, under which the shear strength and unconfined compressive strength of the modified expansive soil were both enhanced. (3) In terms of freeze–thaw durability, the composite fiber-modified soil subjected to freeze–thaw cycles showed significantly lower attenuation rates of shear strength and unconfined compressive strength than the untreated specimens. SEM results showed that local overlapping and bridging structures formed between the fibers and soil particles, which helped delay crack propagation and weaken freeze–thaw damage. Through physical restraint and reinforcement effects, lignin fiber (LF) and polypropylene fiber (PP) jointly improved the mechanical performance and frost resistance of expansive soil, thereby providing an experimental reference for research on fiber modification of expansive soils in seasonally frozen regions.

## 1. Introduction

Expansive soils are widely distributed across 23 provinces, municipalities, and autonomous regions in China, covering a total area of approximately 100,000 km^2^ [1–3]. In some seasonally frozen regions, expansive soils are rich in hydrophilic clay minerals such as kaolinite and illite and therefore exhibit pronounced swelling upon water absorption and shrinkage upon water loss [4–6]. As a result, the soil mass undergoes repeated frost heave and thaw settlement, which further induces the propagation of internal crack networks and structural deterioration [7,8], leading to engineering problems such as slope instability and canal lining damage. Therefore, improving the freeze–thaw stability of expansive soils has become a key technical challenge that must be addressed urgently in cold-region engineering construction [9,10].

To improve the engineering properties of expansive soils, extensive modification studies have been conducted by researchers both in China and abroad. Chemical modification is one of the most widely used methods in engineering practice. It mainly involves the incorporation of additives such as lime, cement, carbide slag, and fly ash to suppress the swell–shrink behavior of soils and enhance their strength and water stability through ion exchange, cementation, and related reactions. For example, Yuan Xiaoqing et al. [11] used carbide slag to improve expansive soil and found that a dosage of 8% effectively inhibited expansion and increased strength; these changes were closely associated with microstructural densification. Hu Qizhi et al. [12] employed a combined modification using blast furnace slag and carbide slag and reported that the hydration products formed could bond soil particles and fill pores. Zhang Yuguo et al. [13] adopted a lime–zeolite powder composite treatment, which further improved the soil strength and stress–strain behavior. Wang Huan et al. [14] investigated the modification effect of alkali-activated low-calcium fly ash on weak expansive soil. Zha Fusheng et al. [15] demonstrated that fly ash and lime–fly ash mixtures could reduce the swell–shrink characteristics of expansive soil. Xu Jiaxiang et al. [16] studied the inhibitory effect and mechanism of desulfurized ash on crack development in expansive soil under wetting–drying cycles. Liu Jingjing et al. [17] investigated the engineering properties and wetting–drying durability of cement–alkali residue-modified expansive soil and revealed the microstructural mechanisms responsible for the improvement in its mechanical properties and durability.

Although chemical modification can effectively improve the engineering properties of expansive soils, it may also pose certain potential environmental impacts. Biological modification is environmentally friendly; however, its solidification effect remains insufficient, it has relatively high requirements for environmental conditions, and its technical maturity is still inadequate, making large-scale engineering application difficult at present. To mitigate these concerns, fiber modification, as a physical reinforcement approach, can compensate for soil defects to some extent and has attracted widespread attention in the field of improved and stabilized soils [18]. The incorporation of fibers can improve the crack resistance and integrity of soils through bridging and reinforcement effects. Duan Junyi et al. [19] carried out triaxial shear tests on shallow expansive soil to investigate the effect of polypropylene fiber on shear strength, and found that fiber length had a significant influence on the shear behavior of the material. Xiao Guiyuan et al. [20] used a sodium silicate–polypropylene fiber composite, which significantly improved soil strength and inhibited crack development under wetting–drying cycles. Seidu et al. [21] used a basalt fiber–lime–high-calcium fly ash ternary stabilization system to improve expansive soil, reducing its plasticity index by 80%, improving the sensitivity of the soil to moisture content changes, and effectively inhibiting its swelling behavior. Sharo AA et al. [22] evaluated the application effect of glass fiber combined with cement for stabilizing expansive soil. The results showed that the incorporation of glass fiber and cement could improve the compressive strength and soaked CBR value of the soil and reduce its swelling tendency. At present, plant fibers have also been widely used in soil improvement because of their environmental friendliness, economy, and renewability [23,24]. Mahyar Arabani et al. [25] enhanced the mechanical properties of bentonite by incorporating wheat fiber, which strengthened the interaction and bonding forces between soil particles. Ji Xinping et al. [26] proposed the composite modification of expansive soil using guar gum and palm fiber, and verified their synergistic effects through water stability tests, unconfined compression tests, and triaxial tests. Zhu Rui et al. [27] examined the improvement effect of lignin fiber on canal-foundation expansive soil under freeze–thaw cycles. Hao Jianbin et al. [28] studied the improvement effect and mechanism of a fly ash–sisal fiber composite on expansive soil. Wang Huan et al. [29] investigated the modification effect of lignin fiber on expansive soil. Through laboratory tests, environmental scanning electron microscopy, and X-ray diffraction analysis, they clarified the corresponding modification mechanism. The improvement mechanism was mainly physical cementation, in which lignin-based cementitious substances filled the soil pores and densified the microstructure without generating new mineral phases; therefore, this process was considered physical modification. Cai Yi et al. [30] investigated the effects of fly ash–lignin fiber composite modification on expansive soil, and found that the incorporation of fly ash not only improved the mechanical properties of expansive soil but also reduced its swell–shrink behavior. Liu et al. [31] also attempted to use bagasse in combination with other materials for expansive soil stabilization. However, plant fibers are water-sensitive, biodegradable, and susceptible to decay, and their long-term durability and mechanical stability may be weakened under harsh environmental conditions. By contrast, synthetic fibers have higher tensile strength and good chemical stability, giving them stronger resistance to severe environments. The combined use of synthetic and plant fibers is expected to integrate the stable physical reinforcement of synthetic fibers with the moisture regulation and pore-filling effects of plant fibers, thereby further improving the comprehensive modification effect on expansive soil. Based on the above findings, fiber modification can effectively enhance the mechanical properties and crack resistance of expansive soil. In particular, polypropylene fiber performs well in restraining soil cracking because of its high tensile strength, whereas lignin fiber, owing to its favorable water absorption and dispersion characteristics, helps regulate moisture distribution within the soil and improve the uniformity of modification. However, studies on the composite modification of expansive soil using polypropylene fiber and lignin fiber remain limited, and in particular, the comprehensive improvement and reinforcement mechanism under the harsh condition of freeze–thaw cycling is still not fully understood.

Therefore, this study focused on expansive soil and employed a polypropylene fiber–lignin fiber system for modification. First, the improvement effects of individual fiber incorporation were analyzed through free swell ratio, no-load swell ratio, and direct shear tests, and composite fiber proportioning tests were then carried out on the basis of the optimized single-fiber results. Subsequently, through freeze–thaw cycle tests, the strength and deformation characteristics of the modified soil were systematically investigated. Combined with scanning electron microscopy (SEM) observations, the microscopic mechanism was analyzed. This study aims to reveal the inhibitory effect of PP and LF fibers on the deterioration of expansive soil properties under freeze–thaw conditions, thereby providing experimental support for research on fiber modification of expansive soils in seasonally frozen regions.

## 2. Materials and methods

### 2.1. Materials

**Fig 1:**
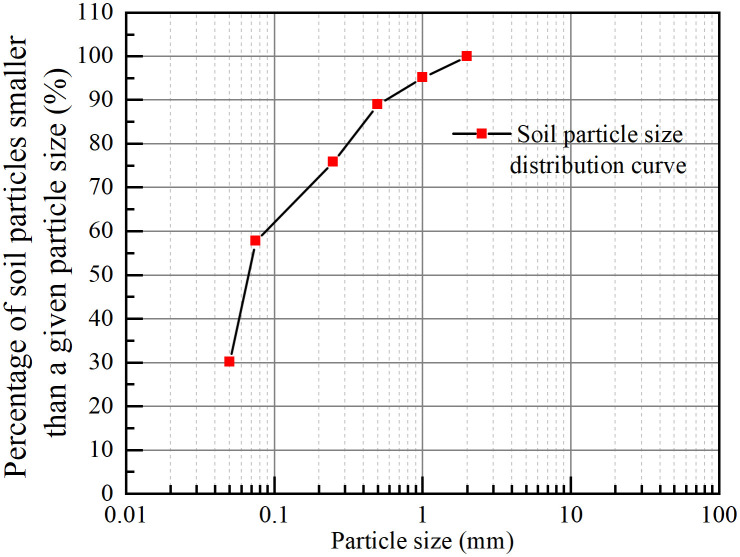
Particle size distribution curve of expansive soil.

The expansive soil used in this study was collected from Hefei, Anhui Province, China, and the soil samples appeared grayish yellow. The basic physical properties and particle composition of the expansive soil samples were determined in accordance with the Standard for Geotechnical Testing Method (GB/T 50123–2019). Before testing, the undisturbed soil samples were naturally air-dried, crushed, and sieved. According to the particle-size distribution test results, soil samples with particle sizes smaller than 2 mm were selected for subsequent tests. The test results showed that the soil contained 43.27% sand, 27.59% silt, and 29.14% clay particles with diameters smaller than 0.005 mm, as presented in Fig 1 and Table 1.

**Table 1:** Physical properties of the expansive soil.

Parameter	Value
Specific gravity	2.72
Liquid limit	59.21%
Plastic limit	19.79%
Plasticity index	39.42
Free swell ratio	52.7%
Maximum dry density	1.65 g/cm^3^
Optimum moisture content	22.0%

**Fig 2:**
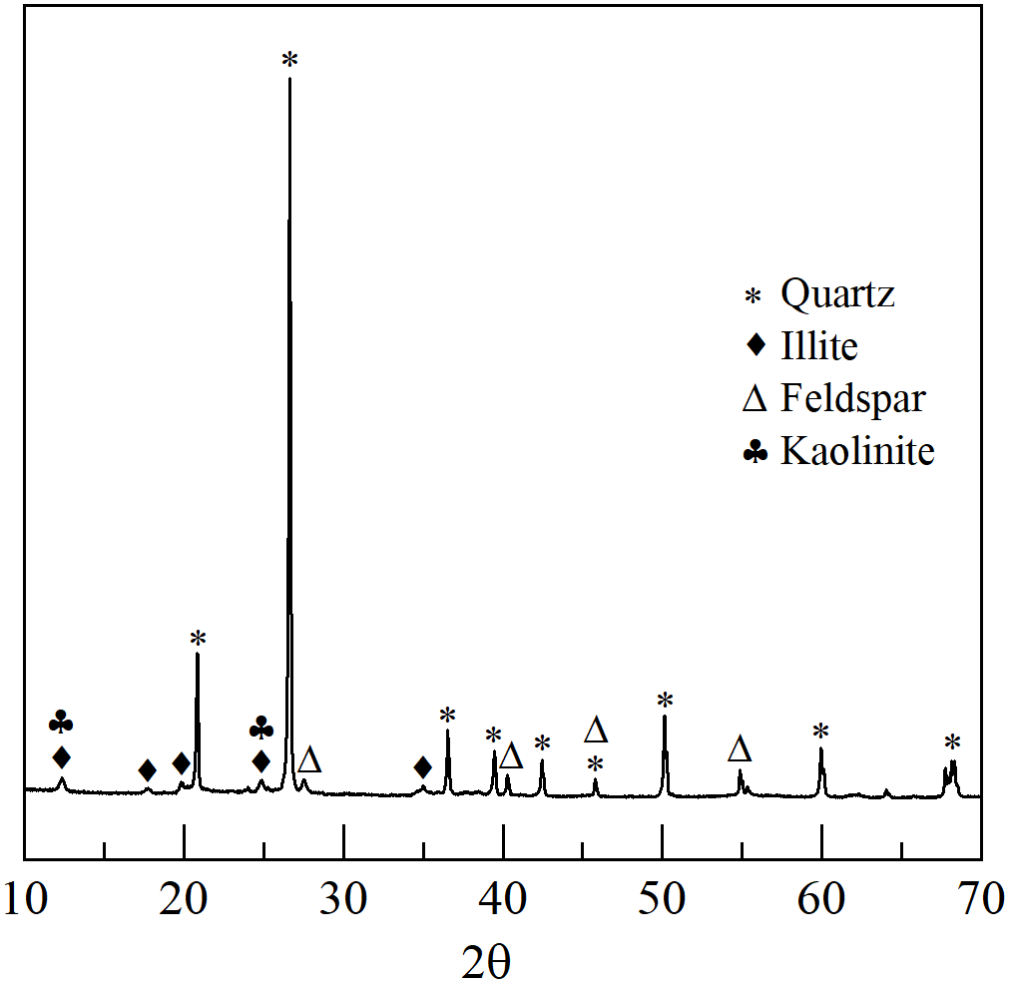
XRD pattern of the untreated expansive soil.

To further clarify the mineral composition of the expansive soil, X-ray diffraction (XRD) tests were conducted on the undisturbed expansive soil, and the results are shown in Fig 2. The XRD pattern indicates that the main identifiable minerals in the test soil include quartz, illite, feldspar, and kaolinite. Among them, the presence of clay minerals such as illite and kaolinite gives the expansive soil relatively high plasticity and water sensitivity.

**Fig 3:**
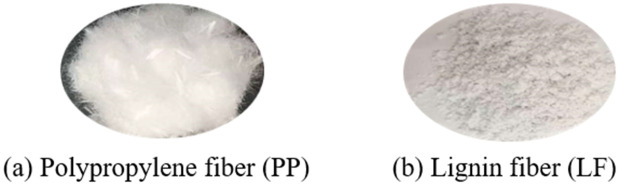
Fiber diagram.

The fibers used in this study were all sourced from Shijiazhuang Chuangsheng Building Materials Technology Co., Ltd. Polypropylene fiber (PP), with a length of 9 mm, an average diameter of 39 μm, an aspect ratio of approximately 231, a breaking strength of 390 MPa, an elastic modulus of 5.3 GPa, and a density of 0.91 g/cm^3^, has high tensile performance and good durability, while also exhibiting low thermal conductivity and relatively stable chemical properties, as shown in Fig 3(a). Lignin fiber (LF) was white and flocculent in appearance, with an original length of approximately 3 mm and a moisture content of no more than 5%. It possesses excellent flexibility and dispersibility and can effectively improve the overall stability, strength, compactness, and uniformity of the stabilized soil, thereby enhancing the comprehensive engineering performance of the material, as shown in Fig 3(b).

### 2.2. Experimental methods

#### 2.2.1. Specimen preparation.

The specific preparation procedure was as follows: first, the expansive soil was crushed, oven-dried, and sieved. With reference to existing studies on fiber-reinforced soils, polypropylene fiber and lignin fiber were then incorporated into the expansive soil at dosages of 0.4%, 0.8%, 1.2%, 1.6%, and 2.0% by dry soil mass, respectively, and mixed uniformly [32]. The standard compaction test results showed that the optimum moisture content of the expansive soil was 22%. To balance the dispersion of fibers in the soil and the forming stability of the specimens, 75% of the optimum moisture content was uniformly selected as the molding moisture content in this study; that is, pure water equivalent to 16.5% of the dry soil mass was added. After mixing, the mixture was sealed and left to stand for 24 h to allow the water to be fully and uniformly distributed within the soil. The specimens were prepared using a layered static compaction method according to different test types: ring-knife specimens with a diameter of 61.8 mm and a height of 20 mm were used for the no-load swell ratio test and direct shear test; cylindrical specimens with a diameter of 39.1 mm and a height of 80 mm were used for the unconfined compressive strength test. Each specimen was compacted in three layers, with an equal mass of mixture added to each layer to ensure uniform internal density. At a moisture content of 16.5%, the target dry density of the specimens was controlled at approximately 1.57 g/cm^3^, corresponding to 95% of the maximum dry density obtained from the standard compaction test. The corresponding wet density was approximately 1.83 g/cm^3^. The wet density was checked by measuring the wet mass and volume of the specimens, and the actual dry density was then calculated according to the molding moisture content, with its deviation from the target dry density controlled within ±2%. After preparation, the specimens were wrapped with plastic film, placed in sealed bags, and cured at 20 ± 2°C and 95% relative humidity for 28 d before subsequent tests.

#### 2.2.2. Test procedure.

In this study, free swell ratio, no-load swell ratio, direct shear, and unconfined compressive strength tests were conducted on fiber-modified expansive soils in accordance with the Standard for Geotechnical Testing Method (GB/T 50123–2019). The freeze–thaw cycle test was carried out with reference to the relevant method in ASTM D560-03 and in combination with the test conditions of this study. Scanning electron microscopy (SEM) testing was performed according to the instrument operating procedures.

Free swell ratio, no-load swell ratio, and direct shear tests were conducted on the soils modified with single-fiber incorporation. (1) Free swell ratio test: The free swell ratio test was carried out as follows: first, the oven-dried and sieved soil sample was transferred into a measuring cylinder through a stemless funnel and then moved into a 30 mL graduated cylinder filled with pure water. After the soil sample had fully absorbed water, expanded, and reached a stable state, the free swell ratio was determined based on the percentage ratio of the volume increment to the initial soil volume. (2) No-load swell ratio test: The no-load swell ratio test was performed as follows: the prepared ring-cutter specimen was first mounted on the measuring device. Water was then continuously added into the soaking tank to maintain the water level approximately 5 mm above the top surface of the specimen. The vertical displacement was monitored in real time using a precision dial indicator. When the fluctuation between two consecutive readings was less than 0.01 mm, the specimen was considered to have reached a stable state. The no-load swell ratio was then calculated as the percentage ratio of the height increment of the ring-cutter specimen after water saturation to its initial height. (3) Direct shear test: For the shear strength test of the single-fiber-modified soil, the specimen was placed in an EDJ-2 strain-controlled direct shear apparatus. The shear tests were conducted at a shear rate of 1 mm/min under different normal stresses of 100, 200, 300, and 400 kPa. The maximum shear stress in the shear stress–shear displacement curve was defined as the peak strength. When the post-peak shear stress entered a relatively stable stage, the stable shear stress was defined as the residual strength. For curves without an obvious post-peak plateau, the stable shear stress near the terminal displacement of the test was taken as the approximate residual strength.

Based on the results of the single-fiber incorporation tests, direct shear strength and unconfined compressive strength tests were conducted on the composite fiber-modified soils. The unconfined compressive strength test was conducted using an RDL-200 universal electronic testing machine at a loading rate of 1 mm/min. The preferred composite fiber proportion was determined according to the mechanical test results.

Then, freeze–thaw durability tests were conducted on the untreated soil and the specimens with the preferred composite fiber proportion, with reference to freeze–thaw conditions used in existing studies on expansive soils and in combination with the test conditions of this study [33]. The test was carried out using a DR-2A freeze–thaw test chamber. In each cycle, the freezing and thawing temperatures were set at −15 ± 2°C and 20 ± 2°C, respectively, and both the freezing and thawing durations were 12 h. The numbers of freeze–thaw cycles were set to 1, 3, 5, 7, and 9. Before the freeze–thaw test, the molding moisture content of the specimens was controlled at 16.5%. During freeze–thaw cycling, the specimens were wrapped with plastic film throughout, and no external drainage channel was provided to reduce moisture loss during the cycles.

**Fig 4:**
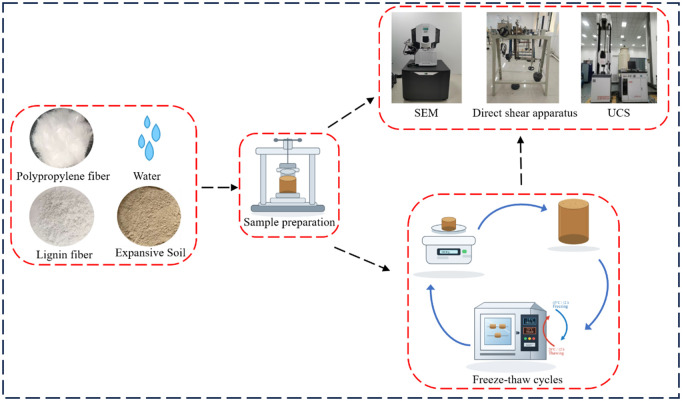
Test procedure.

After the designated number of freeze–thaw cycles was reached, direct shear tests and unconfined compressive strength tests were performed on the untreated soil and composite fiber-modified soil. To ensure the reliability of the test results, three parallel specimens were prepared for each group, and the arithmetic mean was taken as the final result. To qualitatively observe the local microstructural morphology changes of the soil before and after freeze–thaw cycling, representative specimens were selected for SEM tests at a magnification of 500 × . The detailed testing procedure is shown in Fig 4.

## 3. Results and discussion

### 3.1. Free swell ratio of single-fiber-modified soil

**Fig 5:**
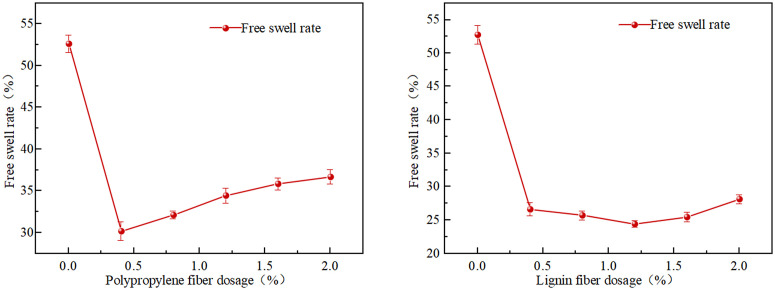
Free swell ratio of modified soil at different fiber types and dosages.

Fig 5 shows the free swell ratio of soils modified with different dosages of single fibers (PP/LF). As can be seen from Fig 5, the incorporation of different fibers effectively reduced the free swell ratio of expansive soil. As further shown in Fig 5(a), when the polypropylene fiber (PP) dosage was 0.4%, the free swell ratio of the modified soil decreased to 30.25%. At this dosage, the specimen no longer exhibited obvious expansibility. Fig 5(b) illustrates the effect of lignin fiber (LF) dosage on the free swell ratio of the specimens. Within the dosage range of 0.4%–1.2%, lignin fiber (LF) markedly reduced the free swell ratio of expansive soil, with the value decreasing from 52.7% to 24.23%. When the dosage exceeded 1.2%, the free swell ratio of the specimen showed an increasing trend, but it remained lower than that of the specimen without fiber addition. This is mainly because, at low dosages, lignin fibers interweave within the expansive soil to form a three-dimensional network structure. During the swelling process of the modified soil, this structure can effectively restrain the soil mass and limit the swelling displacement of soil particles, thereby reducing the free swell ratio. When the fiber dosage exceeded 1.2%, excessive fiber accumulation caused internal interference and entanglement within the soil, which reduced the uniformity and stability of the soil network structure and weakened its restraining effect on the swelling space of the modified soil particles. In terms of reducing the free swell ratio of expansive soil, the improvement rate achieved by polypropylene fiber (PP) was 42.6%, whereas that achieved by lignin fiber (LF) was 54.0%. Therefore, the improvement effect of lignin fiber (LF) was, to some extent, superior to that of polypropylene fiber (PP), and the optimum performance was achieved at an LF dosage of 1.2%.

### 3.2. No-load swell ratio of single-fiber-modified soil

**Fig 6:**
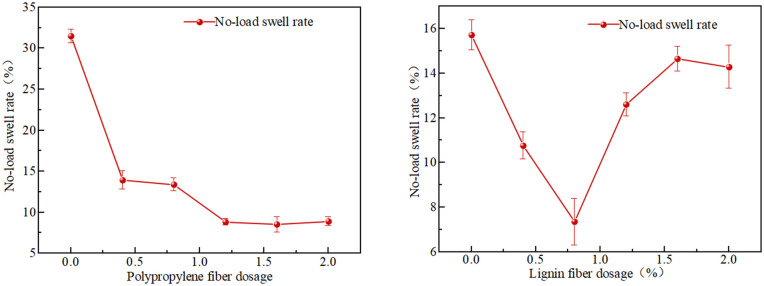
No-load swell ratio of modified soil with different fiber dosages.

Fig 6 presents the no-load swell ratio of soils modified with different fiber dosages. As shown in Fig 6(a), the incorporation of polypropylene fiber (PP) effectively reduced the swelling of expansive soil. Furthermore, with increasing polypropylene fiber (PP) dosage, the no-load swell ratio of expansive soil exhibited a two-stage evolution: it decreased rapidly at the initial stage of fiber addition and then gradually stabilized. In the initial stage, when the fiber dosage ranged from 0.8% to 1.2%, the distributed fiber network effectively restrained soil particle displacement through a spatial confinement effect. However, when the dosage exceeded 1.2%, the stress field distribution gradually approached equilibrium, and further increasing the fiber dosage produced little additional improvement. Fig 6(b) shows the influence of lignin fiber incorporation on the no-load swell ratio of the modified soil. It can be seen that the addition of lignin fiber (LF) reduced the no-load swell ratio of the specimens, and all modified soils exhibited lower values than the untreated expansive soil. With the addition of lignin fiber, the no-load swell ratio first decreased and then increased. This behavior was related to the mechanism by which lignin fiber (LF) acted on expansive soil. Although the three-dimensional network structure of the fiber imposed physical restraint on soil particles, its hydrophilic nature allowed it to absorb water, thereby increasing the swelling potential of the soil. Overall, Fig 6 indicates that within an appropriate dosage range, the incorporation of different single fibers effectively reduced the swell ratio of the modified soil, demonstrating that fiber addition can effectively suppress soil swelling.

**Fig 7:**
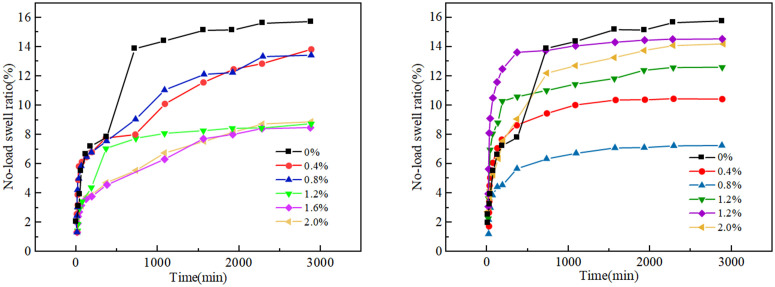
Variation in no-load swell ratio of fiber-modified soil with time.

Fig 7 shows the variation in the no-load swell ratio of modified soils with time after the incorporation of different fibers. The no-load swell ratio of the modified soils with different fiber incorporations exhibited similar time-dependent trends. Specifically, the no-load swell ratio increased gradually with time at the initial stage, and then tended to level off after a certain period, indicating that the specimens almost ceased to undergo further swelling deformation. This behavior can be attributed to the fact that, at the initial stage, the particles within the specimens were strongly affected by the surrounding environment and thus exhibited pronounced swelling. As time progressed, the internal particles of the modified soil gradually became stable, causing the curves to flatten accordingly.

### 3.3. Shear strength of single-fiber-modified soil

Tables 2 and 3 present the shear strength of the soils under different normal stresses. The shear strength of the soil was closely related to the applied normal stress. For both untreated expansive soil and modified soil, the shear strength of the specimens gradually increased with increasing normal stress. The application of shear force induced compressive deformation in different layers of the soil, which in turn caused displacement differences between soil layers. Meanwhile, as the normal stress increased, the interparticle bonding within the specimen was gradually enhanced, thereby leading to a progressive increase in shear strength.

**Table 2:** Shear strength of polypropylene fiber-modified soil under different normal stresses.

Normal stress (KPa)	Shear strength under different PP fiber dosages (KPa)					
	No fiber addition	0.4%	0.8%	1.2%	1.6%	2.0%
100	85.5	101.98	124.84	138.03	102.86	98.47
200	102.75	138.03	168.8	165.28	121.33	131.88
300	139.5	163.66	217.51	185.28	161.77	158.25
400	162.15	189.9	251.44	232.1	219.79	218.03

**Table 3:** Shear strength of lignin fiber-modified soil under different normal stresses.

Normal stress (KPa)	Shear strength at different LF dosages (KPa)					
	No fiber addition	0.4%	0.8%	1.2%	1.6%	2.0%
100	85.5	116.05	114.29	116.05	96.7	110.78
200	102.75	156.49	167.04	153.85	139.79	148.03
300	139.5	182.87	177.59	251.33	164.40	212.54
400	162.15	208.37	219.79	293.64	223.84	295.4

Furthermore, with increasing single-fiber dosage, the shear strength of the specimens generally showed a trend of first increasing and then decreasing. The incorporation of a single fiber could effectively improve the shear strength of the soil, indicating that an optimum fiber dosage exists. Compared with the untreated expansive soil, after the addition of lignin fiber (LF), the maximum increase in soil shear strength reached 82.17%, whereas the maximum increase achieved by polypropylene fiber (PP) was 64.28%. For the specimens containing lignin fiber (LF), when the fiber dosage was 1.2%, the shear mechanical parameters were significantly higher than those of specimens with other fiber dosages. This indicates that a lignin fiber (LF) dosage of 1.2% can effectively improve the shear mechanical parameters of the specimens.

To quantitatively characterize the influence of single-fiber dosage on shear strength parameters, the Mohr–Coulomb criterion, $\tau_{f}=c+\sigma_{n}\text{tan}\varphi$, was used to linearly fit the shear strength–normal stress data in Tables 2 and 3. The fitted intercept was used to represent the cohesion c, and the internal friction angle φ was back-calculated from the fitted slope. The results are shown in Table 4.

**Table 4:** Fitted shear strength parameters for soils with different single-fiber dosages.

Soil type	Fitting relationship	R^2^	Cohesion c/kPa	Internal friction angle φ/°
untreated soil	y = 0.27x+55.8	0.982	55.8	15.11
0.4%LF	y = 0.30x+90.1	0.986	90.1	16.7
0.8%LF	y = 0.33x+87.92	0.947	87.92	18.26
1.2%LF	y = 0.63x+46.16	0.968	46.16	32.21
1.6%LF	y = 0.41x+54.69	0.975	54.69	22.3
2.0%LF	y = 0.62x+37.10	0.973	37.10	31.8
0.4%PP	y = 0.29x+76.05	0.993	76.05	16.17
0.8%PP	y = 0.43x+83.52	0.995	83.52	23.27
1.2%PP	y= 0.30x+104.62	0.967	104.62	16.7
1.6%PP	y = 0.39x+53.03	0.951	53.03	21.31
2.0%PP	y = 0.39x + 55.40	0.967	55.40	21.06

The fitting results show that the R^2^ values of all groups ranged from 0.947 to 0.995, indicating good fitting performance. This suggests that the obtained c and φ parameters can be used to evaluate the variation in shear strength parameters under different fiber dosages. When the PP fiber dosage was 1.2%, the cohesion reached its maximum value of 104.62 kPa, indicating that an appropriate amount of PP fiber could improve the cohesive capacity of the specimens through fiber bonding, particle restraint, and local reinforcement. The internal friction angle of the specimen with 0.8% PP was relatively large, suggesting that interparticle friction, interlocking, and fiber–soil interface friction contributed more significantly at this dosage. When the PP dosage continued to increase, the cohesion and internal friction angle did not continue to increase simultaneously, which may be related to increased difficulty in fiber dispersion, local agglomeration, or pore disturbance. When the LF fiber dosage was 1.2%, the internal friction angle reached 32.21°, while the cohesion was relatively low, indicating that the improvement in shear strength at this proportion was mainly reflected in enhanced frictional resistance, particle interlocking, and fiber bridging under high normal confinement. Combined with the data in the table, it can be seen that PP fiber at an appropriate dosage is more beneficial for improving the apparent cohesion of the soil, whereas LF fiber shows a more pronounced effect on improving the internal friction angle.

### 3.4. Mix proportion of composite fiber modification

In this study, the composite fiber dosage refers to the sum of the lignin fiber (LF) and polypropylene fiber (PP) dosages. Based on the single-fiber incorporation study, the lignin fiber (LF) dosage was fixed at 1.2%, and the mix proportions of composite fiber modification with different polypropylene fiber (PP) dosages (0.4%, 0.8%, 1.2%, and 2.0%) were investigated.

#### 3.4.1. Shear strength of composite fiber-modified soil.

Table 5 presents the shear strength of composite fiber-modified soil under different normal stresses. At the same fiber dosage, the shear strength of the modified soil specimens gradually increased with increasing normal stress, mainly because the applied normal stress restrained specimen deformation. Furthermore, under the same normal stress, as the polypropylene fiber (PP) dosage increased, the shear strength of the modified soil generally exhibited a trend of first increasing and then decreasing. Among the tested mixtures, the modified soil containing 1.2% LF and 1.2% PP showed peak shear stresses of 139.761 kPa, 178.437 kPa, 223.266 kPa, and 244.362 kPa under different normal stresses, respectively. Under different normal stress conditions, the composite fiber proportion of 1.2% LF and 1.2% PP showed the lowest average deviation relative to the maximum value across all working conditions, indicating that this proportion exhibited relatively stable overall performance.

**Table 5:** Shear strength of composite fiber-modified soil under different normal stresses.

Compound blending ratio	Shear strength under different normal stresses (KPa)			
	100	200	300	400
1.2% LF + 0.4%PP	116.907	153.825	175.8	212.718
1.2%LF + 0.8%PP	122.181	163.494	204.807	258.426
1.2% LF + 1.2%PP	139.761	178.437	223.266	244.362
1.2% LF + 1.6%PP	138.882	172.284	204.807	245.241
1.2% LF + 2.0%PP	140.64	182.832	214.693	232.935

According to the fitting results in Table 6, the fitting coefficients R^2^ of the shear strength envelopes for different composite proportions ranged from 0.971 to 0.997, indicating good fitting performance. Further comparison of the fitted parameters shows that although the 1.2% LF + 1.2% PP proportion did not achieve the maximum shear strength under all normal stresses, its cohesion c remained at a relatively high level, its internal friction angle φ was maintained at approximately 20°, and its strength increased relatively steadily within the normal stress range of 100–400 kPa. This indicates that this proportion provided favorable particle bonding, fiber bridging, and interface friction effects.

**Table 6:** Fitted shear strength parameters for soils with different composite fiber proportions.

Soil type	Fitting relationship	R^2^	Cohesion c/kPa	Internal friction angle φ/°
1.2%LF + 0.4%PP	y=0.31x+87.46	0.991	87.46	17.19
1.2%LF + 0.8%PP	y=0.45x+74.72	0.996	74.72	24.23
1.2%LF + 1.2%PP	y=0.36x+106.80	0.981	106.80	19.73
1.2%LF + 1.6%PP	y=0.35x+102.40	0.997	102.40	19.37
1.2%LF + 2.0%PP	y=0.31x+115.59	0.971	115.59	17.16

By contrast, although the 1.2% LF + 2.0% PP proportion exhibited relatively high fitted cohesion, its internal friction angle decreased, and its shear strength under a normal stress of 400 kPa was lower than those of the 1.2% LF + 0.8% PP and 1.2% LF + 1.6% PP proportions, indicating that its strength was not fully mobilized under high normal stress. This may be related to the increased difficulty of PP fiber dispersion and fiber agglomeration at excessive PP fiber dosage, which weakened particle interlocking and interface friction. Considering shear strength, cohesion, internal friction angle, and strength stability under different normal stresses, 1.2% LF + 1.2% PP was selected as the composite fiber proportion with relatively better overall performance within the test range of this study.

#### 3.4.2. Compressive strength of composite fiber-modified soil.

**Fig 8:**
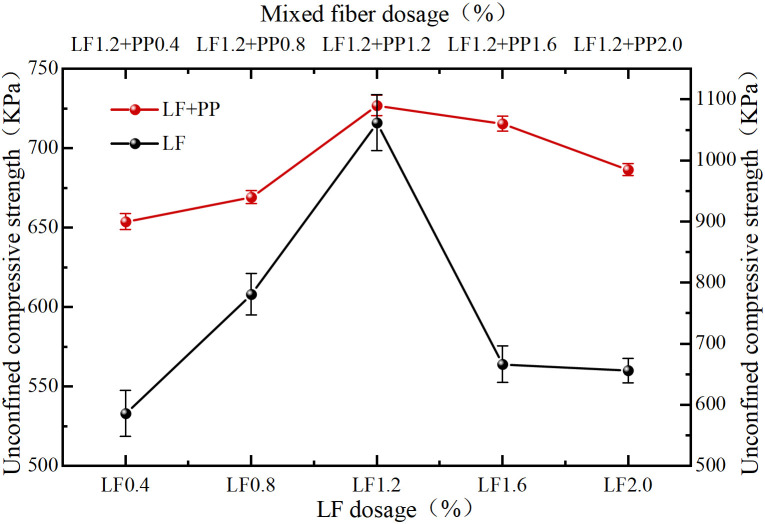
Unconfined compressive strength of specimens at different fiber dosages.

Fig 8 presents the unconfined compressive strength of fiber-modified soils. When lignin fiber (LF) was added alone, the unconfined compressive strength of the modified soil first increased and then decreased with increasing fiber dosage. When the dosage increased from 0.4% to 1.2%, the compressive strength of the specimen increased by 34.51%. However, when the dosage exceeded 1.2%, the mechanical performance of the material decreased markedly. Specifically, when the dosage was 1.6%, the strength decreased by 21.33%, whereas at a dosage of 2.0%, the strength loss reached 21.87%. These results indicate that the material has a distinct critical dosage threshold, and the unconfined compressive strength reached its peak at an LF dosage of 1.2%.

Furthermore, when the lignin fiber (LF) dosage was fixed at 1.2%, the unconfined compressive strength of the specimens also showed a trend of first increasing and then decreasing with increasing polypropylene fiber (PP) dosage. Under the combined action of 1.2% LF and 1.2% PP, the unconfined compressive strength of the specimen reached its maximum value. The unconfined compressive strength of the specimens with composite fiber incorporation was overall higher than that of specimens with single-fiber addition, indicating that the performance under composite fiber modification was superior to that under single-fiber modification. This can be attributed to the fact that the incorporation of composite fibers optimized the internal network structure of the expansive soil. The ductility of polypropylene fiber effectively compensated for the brittleness of lignin fiber, while the two fibers with different aspect ratios formed a complementary reinforcement network with a continuous structure.

### 3.5. Freeze–thaw characteristics of composite fiber-modified soil

Based on the results of the previous conventional mechanical tests, the combination of 1.2% lignin fiber (LF) and 1.2% polypropylene fiber (PP) was selected as the object of the freeze–thaw cycle test in this study. On the basis of this proportion, the freeze–thaw characteristics of the modified soil were further investigated.

#### 3.5.1. Dimensional changes of soil under freeze–thaw cycles.

**Fig 9:**
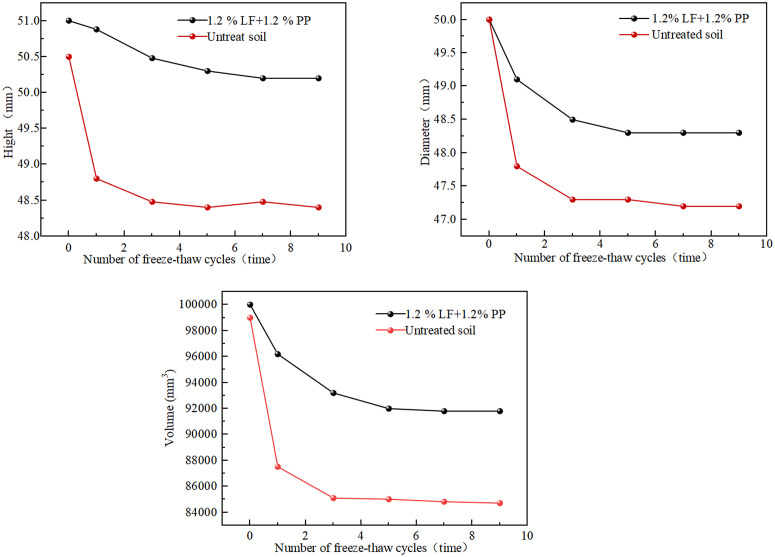
Evolution of dimensional parameters of modified soil with increasing freeze–thaw cycles.

Fig 9 shows the variation in the height, diameter, and volume of the untreated and modified soil specimens during freeze–thaw cycling. As shown in the figure, the dimensional deterioration of the modified soil exhibited a typical three-stage characteristic. The first stage was the rapid change stage, which mainly occurred during the first cycle, during which the height, diameter, and volume of the specimens all changed markedly. The second stage was the adjustment stage, which mainly occurred from the 1st to the 5th cycle, during which the magnitude of dimensional change was significantly smaller than that in the first stage. The third stage was the stable change stage. After five freeze–thaw cycles, the dimensional change of the specimens tended to become gradual, indicating that the internal structure of the specimens had completed a process of self-organized optimization.

As shown in Fig 9(a), the height of the untreated soil changed markedly after freeze–thaw cycling, indicating that moisture migration and ice crystal growth during freeze–thaw cycles caused vertical structural disturbance in the soil. In contrast, the modified soil showed a smaller height variation, suggesting that the fiber network formed by lignin fiber (LF) and polypropylene fiber (PP) could exert spatial constraint on soil particles, thereby weakening the vertical deformation induced during freeze–thaw cycling. As shown in Fig 9(b), the diameter of the specimens gradually decreased with increasing freeze–thaw cycles. However, the diameter change of the modified soil was smaller than that of the untreated soil, indicating that the overlapping and bridging effects of fibers inside the soil could limit lateral particle displacement and improve the overall stability of the specimens. As shown in Fig 9(c), the volume change of the specimens was affected by the combined changes in height and diameter. The volumetric shrinkage ratio of the modified soil in the first freeze–thaw cycle was 3.80%, which was 67.5% lower than the 11.68% observed for the untreated soil. After nine cycles, the cumulative volumetric shrinkage ratio of the modified soil was only 56.4% of that of the untreated soil, indicating that the freeze–thaw deformation resistance of the specimens was significantly enhanced after fiber modification. This is because, during repeated freeze–thaw cycling of the untreated soil, internal water repeatedly froze and thawed, and ice crystals pushed soil particles to rearrange, gradually forming internal pores and cracks. In the composite fiber-modified soil, the combined effects of polypropylene fiber bridging and lignin fiber regulation of internal moisture inhibited the damage of the soil under freeze–thaw cycles.

#### 3.5.2. Shear stress–displacement curves of soil under freeze–thaw cycles.

**Fig 10:**
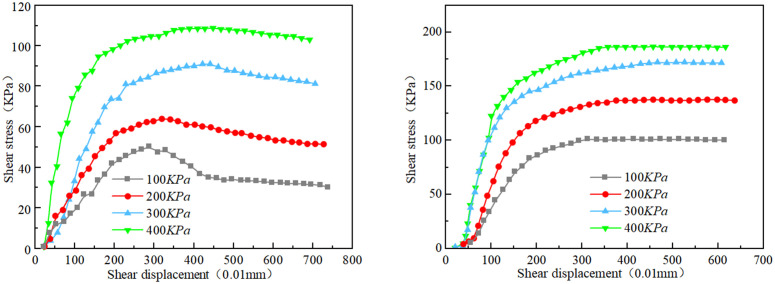
Shear stress–displacement curves of specimens under freeze–thaw cycles.

Fig 10 shows the shear stress–displacement curves of the specimens after nine freeze–thaw cycles under different normal stresses, where Fig 10(a) represents untreated expansive soil and Fig 10(b) represents composite fiber-modified soil. As can be seen from the figure, the curves of the two types of specimens exhibited similar variation characteristics. With the progress of loading, the shear stress–displacement curves exhibited a two-stage response, first increasing rapidly and then gradually tending to stabilize. In the initial loading stage, the shear stress increased rapidly with increasing shear displacement. At this stage, the interactions among internal particles and the resistance to deformation were relatively strong, and therefore a pronounced stress change was observed under small deformation conditions. As the displacement continued to increase, the shear stress–displacement curves gradually became flatter. This was mainly because, as loading proceeded, more internal damage developed, the bonding condition between particles changed, and a sliding tendency emerged along the shear plane, eventually leading to shear failure of the specimen. Comparison between Fig 10(a) and Fig 10(b) shows that, compared with the untreated soil, the modified soil after freeze–thaw cycling exhibited a more obvious stress increase, and its peak stress under different normal stresses was generally higher than that of the untreated soil, indicating stronger shear resistance and deformation coordination capacity. This is because the network structure formed by the composite fibers and their moisture-regulating effect could better limit the displacement variation of soil particles and control moisture distribution, thereby maintaining the bonding and shear resistance between soil particles.

#### 3.5.3. Variation in soil shear strength under freeze–thaw cycles.

**Fig 11:**
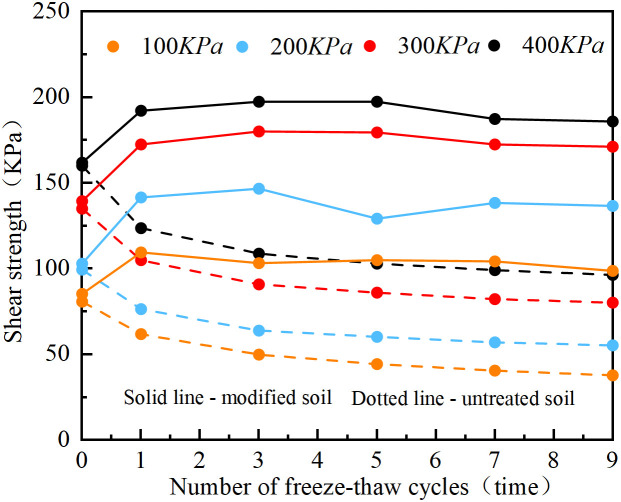
Shear strength of specimens under different numbers of freeze–thaw cycles.

Fig 11 illustrates the variation in soil shear strength with different numbers of freeze–thaw cycles. The shear strength of untreated soil and modified soil exhibited different trends with increasing freeze–thaw cycles. For untreated soil, the shear strength of the specimens gradually decreased as the number of freeze–thaw cycles increased, indicating that natural expansive soil was strongly affected by freeze–thaw cycling. In contrast, the shear strength of the expansive soil modified with composite fibers showed a trend of first increasing and then stabilizing. This indicates that fiber addition can effectively resist the adverse effects of low temperature, and that the combined incorporation of polypropylene fiber (PP) and lignin fiber (LF) can effectively improve the frost resistance of expansive soil. It can also be seen from the figure that the shear strength of the improved soil specimens was higher than that of the untreated soil specimens under all freeze–thaw cycle conditions. This demonstrates that the combined effect of composite fibers can effectively enhance the shear strength of the improved soil.

As shown in Tables 7 and 8, the fitting results of the shear strength of untreated soil and 1.2% LF + 1.2% PP modified soil under different numbers of freeze–thaw cycles are presented, and all fitted R^2^ values were above 0.97. To more intuitively observe the modification effect of composite fiber-modified soil under freeze–thaw cycling, shear strength envelope diagrams were further plotted. Overall, the cohesion and internal friction angle of both types of soil specimens decreased with increasing freeze–thaw cycles. For the untreated soil, the fitted cohesion decreased from 55.80 kPa after 0 cycles to 17.815 kPa after 9 cycles, with an attenuation rate of 68.07%; the internal friction angle decreased from 14.93° to 11.22°, with an attenuation rate of 24.85%. For the modified soil, the cohesion decreased from 106.80 kPa to 41.56 kPa, with an attenuation rate of 61.09%; the internal friction angle decreased from 19.73° to 16.57°, with an attenuation rate of 16.02%.

**Table 7:** Fitted shear strength parameters of untreated soil under different freeze–thaw cycles.

Number of cycles	Fitting relationship	R^2^	Cohesion c/kPa	Internal friction angle φ/°
0cycles	y=0.2667x+55.8	0.974	55.8	14.93
1cycles	y=0.2135x+38.215	0.976	38.215	12.05
3 cycles	y=0.2029x+27.585	0.981	27.585	11.47
5 cycles	y=0.2014x+23.04	0.987	23.04	11.39
7 cycles	y=0.2002x+19.75	0.989	19.75	11.32
9 cycles	y=0.1984x+17.815	0.989	17.815	11.22

**Table 8:** Fitted shear strength parameters of modified soil under different freeze–thaw cycles.

Number of cycles	Fitting relationship	R^2^	Cohesion c/kPa	Internal friction angle φ/°
0cycles	y=0.3586x+106.80	0.981	106.8	19.73
1cycles	y=0.3340x+75.305	0.996	75.305	18.47
3 cycles	y=0.3184x+60.145	0.997	60.145	17.66
5 cycles	y=0.3020x+50.645	0.995	50.645	16.80
7 cycles	y=0.2895x+45.525	0.995	45.525	16.15
9 cycles	y=0.2975x+41.56	0.996	41.56	16.57

**Fig 12:**
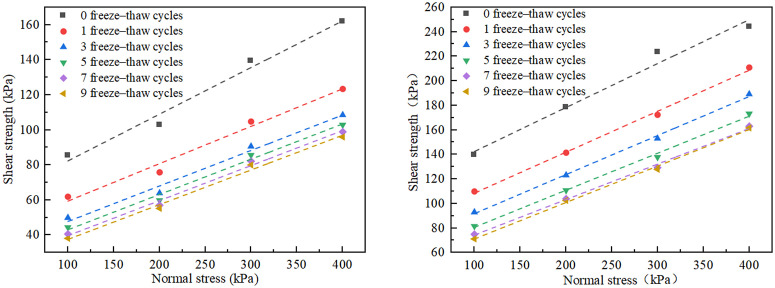
Fitted shear strength curves of different soils under freeze–thaw cycles.

As shown in Fig 12, with increasing freeze–thaw cycles, the fitted strength curves of both types of soil shifted downward overall, indicating continuous attenuation of cohesion c. Meanwhile, the change in curve slope was relatively small, suggesting that φ was less affected by freeze–thaw cycling than c. Compared with the untreated soil, the curves of the composite fiber-modified soil were located at higher positions under all freeze–thaw cycles and still maintained higher intercepts and slopes after 9 cycles. This indicates that the bridging network and filling effect formed by the composite fibers could better envelop soil particles and enhance interfacial friction, thereby delaying cohesion loss and the penetration of the shear plane. Therefore, the modified soil exhibited higher residual shear resistance.

#### 3.5.4. Stress–strain curves of expansive soil under freeze–thaw cycles.

**Fig 13:**
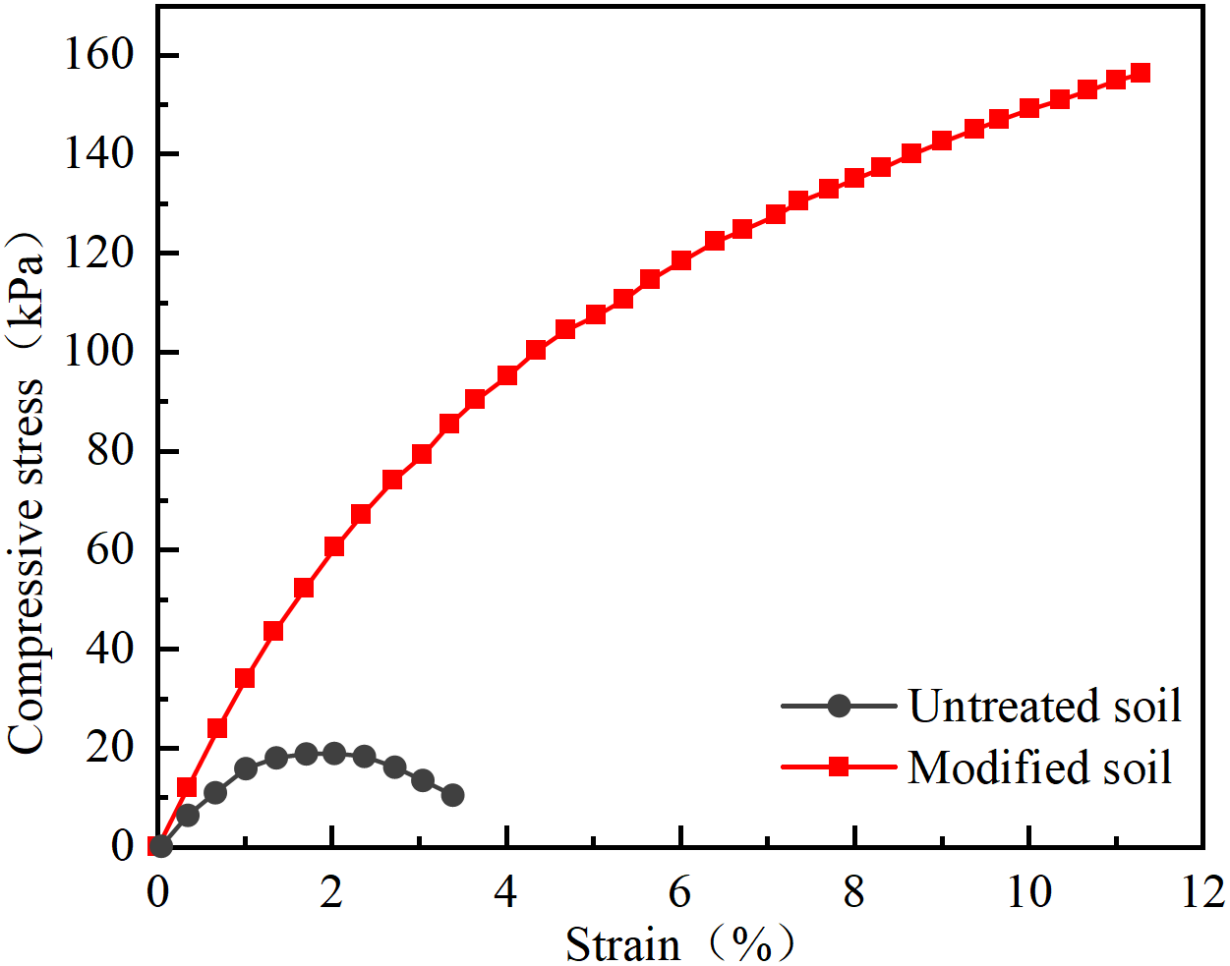
Comparison of stress–strain curves of specimens after 9 freeze–thaw cycles.

Fig 13 compares the stress–strain curves of expansive soil and modified soil, taking the case of nine freeze–thaw cycles as an example. The overall stress–strain response of expansive soil exhibited a strain-softening pattern. The curve can be divided into three stages: the elastic ascending stage, the plastic ascending stage, and the failure stage. At the initial stage of axial compression, the expansive soil specimen showed relatively high load-bearing capacity, and the stress increased rapidly with an approximately linear trend. As surface microcracks initiated and developed, the rate of stress increase gradually slowed, accompanied by plastic deformation. After the peak stress was reached, the cracks within the specimen gradually interconnected, and the stress then decreased rapidly. The resulting failure cracks extended downward along the diagonal from the top to the bottom of the specimen. Furthermore, at the initial stage of increasing strain, the stress of the modified soil rose rapidly and then gradually tended to stabilize. The overall stress–strain curve of the modified soil exhibited a strain-hardening pattern. A comparison of the stress–strain curves of the modified soil and expansive soil shows that the deformation resistance of the modified soil was significantly greater than that of the untreated soil.

**Fig 14:**
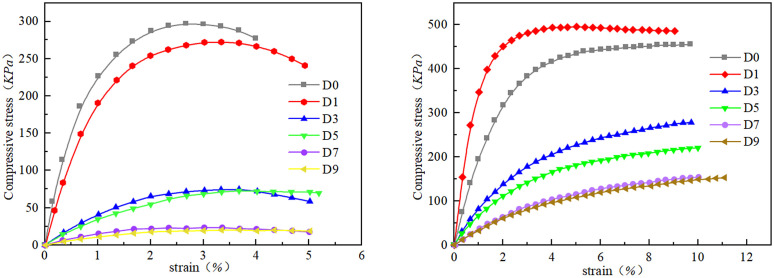
Stress–strain curves of soils after different numbers of freeze–thaw cycles.

Fig 14 shows the stress–strain curves of expansive soil and modified soil under freeze–thaw cycles. As can be seen from the figure, with the increase in the number of freeze–thaw cycles, the variation trend of the stress–strain curves of both expansive soil and modified soil gradually became gentler. This indicates that different numbers of freeze–thaw cycles affected the stress–strain response of the specimens. As the number of freeze–thaw cycles increased, the axial stress–strain curve of expansive soil became flatter and its bearing capacity was relatively low, whereas the modified soil retained a certain level of bearing capacity, indicating that fiber incorporation improved the freeze–thaw performance of expansive soil.

#### 3.5.5. Compressive strength of specimens under freeze–thaw cycles.

**Fig 15:**
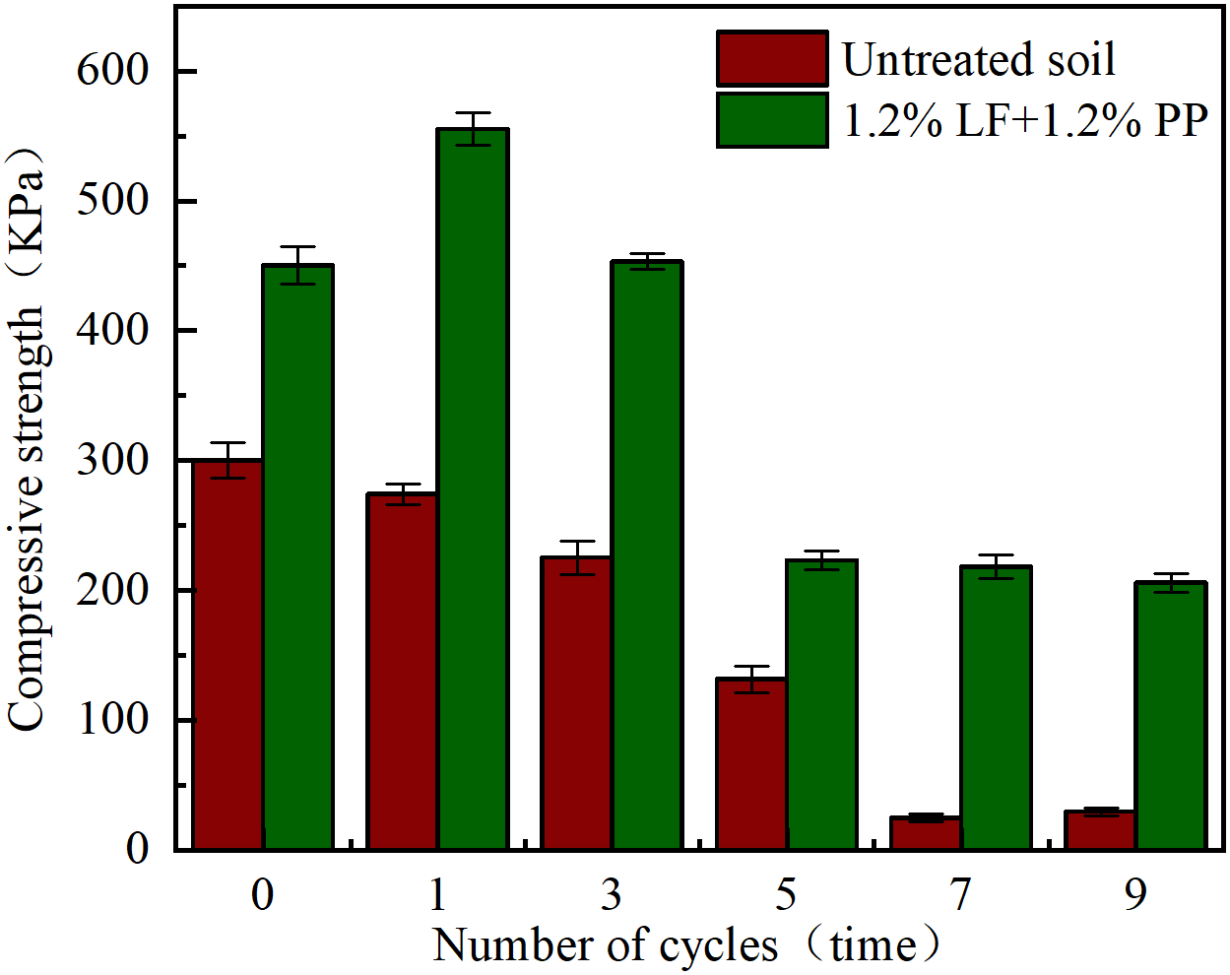
Compressive strength of specimens under freeze–thaw cycles.

Fig 15 compares the strength of expansive soil and modified soil under freeze–thaw cycles. As can be seen from the figure, the compressive strength of expansive soil specimens showed a decreasing trend with increasing freeze–thaw cycles. For the modified soil specimens, the strength first increased and then decreased as the number of freeze–thaw cycles increased. The increase in compressive strength after the first freeze–thaw cycle may be attributed to the fact that freeze–thaw action enhanced the friction and cohesion between the reinforcing materials and the soil. During freezing, soil expansion may have subjected the reinforcing materials to a certain tensile stress, thereby increasing the degree of contact between the reinforcing materials and the soil. After thawing, this close contact state was maintained, allowing the reinforcing materials in the reinforced soil to better mobilize their tensile strength under compression, restrain soil deformation, and thereby improve the unconfined compressive strength. However, after multiple freeze–thaw cycles, this effect gradually weakened or even disappeared. With further increases in freeze–thaw cycles, the compressive strengths of both expansive soil and modified soil specimens gradually tended to stabilize. Further analysis showed that the compressive strength of expansive soil decreased by 93.29% with increasing freeze–thaw cycles, whereas that of the modified soil decreased by 65.49%, indicating that fiber incorporation effectively inhibited the freeze–thaw deterioration of expansive soil. Under different numbers of freeze–thaw cycles, the compressive strength of the modified soil remained higher than that of expansive soil, indicating that the modified soil exhibited better post-freeze–thaw compressive performance than the natural expansive soil.

#### 3.5.6. Microstructural characteristics of expansive soil under freeze–thaw cycles.

**Fig 16:**
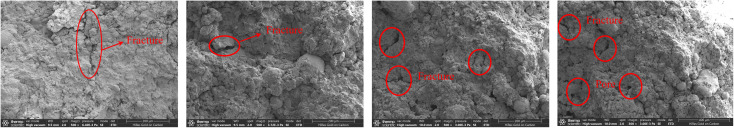
Microstructure of expansive soil under different numbers of freeze–thaw cycles (×500).

Fig 16 shows the microstructural evolution of untreated soil under different numbers of freeze–thaw cycles. In its original state, the untreated soil already exhibited a certain number of micropores and cracks. During the solid–liquid phase transition process, repeated freeze–thaw cycles led to gradual enlargement of internal pores, an increase in the number of cracks, and the formation of cavities and crack propagation caused by the extrusion of surrounding soil particles due to ice crystal growth, which disrupted interparticle bonding and gradually loosened the soil structure. Together with the macroscopic changes in specimen dimensional parameters and the decreases in shear and compressive strengths, these results indicate that the cumulative damage caused by pore enlargement and crack development under freeze–thaw cycles is an important reason for the attenuation of mechanical properties.

**Fig 17:**
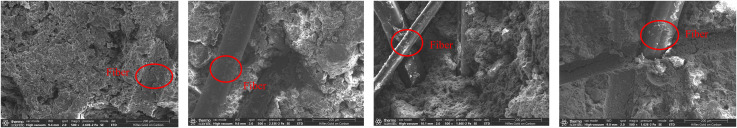
Microstructure of modified soil after different numbers of freeze–thaw cycles (×500).

Fig 17 shows the microstructural evolution of the modified soil under different numbers of freeze–thaw cycles. Compared with the untreated soil (Fig 16), with increasing freeze–thaw cycles, the development of pores and cracks in the modified soil was relatively delayed, and the local structural integrity remained better. Locally overlapping fibers distributed near cracks and bridging structures between fibers and soil particles could be observed, which may limit the displacement of soil particles during freeze–thaw cycling. The water absorption of lignin fiber may induce pre-swelling and fill part of the pores between soil particles, while reducing the amount of water that can freeze into ice crystals and weakening the extrusion of soil particles by ice crystals. Polypropylene fiber can span cracks and exert a bridging effect, thereby delaying the propagation of some cracks. Under the combined action of the two fibers, the changes in specimen dimensional parameters were weakened, allowing the soil to maintain better stability during frost heave and thaw settlement.

## 4. Conclusions

This study analyzed the modification mechanisms and freeze–thaw durability of lignin fiber (LF) and polypropylene fiber (PP) in expansive soil. The main conclusions are as follows.

(1) When lignin fiber (LF) or polypropylene fiber (PP) was incorporated individually, both fibers improved the engineering properties of expansive soil. Within the tested dosage range, LF produced the larger reduction in free swell ratio and was more prominent in improving shear strength. At an LF dosage of 1.2%, the free swell ratio was significantly reduced and the shear strength was markedly increased.
(2) In this study, the composite proportion of 1.2% LF + 1.2% PP achieved a good modification effect. Both the unconfined compressive strength and direct shear strength of the modified soil were significantly improved. The fiber network formed by the composite fibers optimized the soil structure, thereby enhancing the strain coordination capacity of the soil.
(3) For the selected 1.2% LF + 1.2% PP mixture, after freeze–thaw cycles, the cumulative volumetric shrinkage ratio of the modified soil was only 56.4% of that of the untreated soil, and its compressive strength attenuation rate was 27.8 percentage points lower than that of natural expansive soil. This indicates that the incorporation of composite fibers effectively improved the freeze–thaw performance of expansive soil. SEM microstructural characterization showed that fiber overlapping and bridging could restrain soil particle displacement and delay the development of frost-heave-induced cracks. The mechanical anchoring effect of polypropylene fiber and the water absorption characteristics of lignin fiber jointly reduced the damaging effect of ice crystal growth.
(4) The incorporation of composite fibers can improve the mechanical properties and freeze–thaw stability of expansive soil, showing certain application potential. However, the field mixing dispersibility, compaction quality control, unit treatment cost, and long-term environmental impact of PP fibers still require further evaluation.
(5) Owing to the limitations of laboratory test conditions, this study mainly evaluated the initial swelling behavior, mechanical properties, and freeze–thaw stability of composite fiber-modified expansive soil. The shrinkage deformation and crack evolution under wetting–drying cycles have not yet been systematically analyzed. In future work, the shrinkage limit, volumetric shrinkage ratio, wetting–drying cyclic deformation, and quantitative crack characterization can be combined to further evaluate its long-term cyclic swell–shrink durability.
